# Abnormal FDG-PET Patterns and Amyloid Positivity Predict Personality Changes in Cognitively Preserved Individuals

**DOI:** 10.3390/brainsci16080812

**Published:** 2026-07-30

**Authors:** Cristelle Rodriguez, Marie-Louise Montandon, Valentina Garibotto, Sven Haller, François R. Herrmann, Panteleimon Giannakopoulos

**Affiliations:** 1 Medical Direction, Geneva University Hospitals, 1205 Geneva, Switzerland; cristelle.rodriguez@hug.ch; 2 Department of Rehabilitation and Geriatrics, Geneva University Hospitals and University of Geneva, 1205 Geneva, Switzerland; marie.montandon@unige.ch (M.-L.M.);; 3 Diagnostic Department, Division of Nuclear Medicine and Molecular Imaging, Geneva University Hospitals and University of Geneva, 1205 Geneva, Switzerland; valentina.garibotto@hug.ch; 4 CIRD Centre d’imagerie Rive Droite in Geneva, 1205 Geneva, Switzerland; sven.haller@me.com; 5 Department of Surgical Sciences, Radiology, Uppsala University, 75309 Uppsala, Sweden; 6 Department of Neuroradiology, Faculty of Medicine, University of Geneva, 1205 Geneva, Switzerland; 7 Department of Psychiatry, University of Geneva, 1205 Geneva, Switzerland; 8 Geneva Office of Health, 1205 Geneva, Switzerland

**Keywords:** imaging markers, personality, normal aging, Alzheimer’s disease

## Abstract

**Highlights:**

**What are the main findings?**

- FDG PET hypometabolism is associated with the progressive decrease in openness prior to the emergence of clinically overt dementia.
- Amyloid positivity is related to the decrease in agreeableness in old age.

**What are the implications of the main findings?**

- Personality factors may not only contribute to the emergence of AD pathology but also change in the presence of AD-related PET abnormalities.
- Cerebrovascular burden does not lead to significant personality changes in elderly controls.

**Abstract:**

**Background/Objectives**: Clinically overt Alzheimer’s disease (AD) is associated with significant changes in personality factors. Earlier studies indicated that personality factors do not remain stable in elderly controls. Whether the early presence of AD neuroimaging markers may predict subsequent changes in personality factors is still debatable. **Methods**: To address this issue, we examined the association between Big Five factor scores and baseline AD and vascular imaging markers in a previously established cohort of 58 elderly controls with a 4.5-year follow-up. Personality was assessed with the Neuroticism–Extraversion–Openness Personality Inventory—Revised scale at inclusion and 55-month follow-up. Regression models were used to identify predictors of personality factor changes including time, age, sex, APOE epsilon 4 allele, baseline MMSE scores, amyloid PET positivity, abnormal FDG-PET patterns, mesial temporal lobe atrophy and Fazekas scores, and number of microbleeds. **Results**: In both univariate and multivariable models, the decrease in openness was related to abnormal FDG PET patterns. This single variable explained 15% of the variance of this personality factor. In univariate models, lower MMSE scores and amyloid positivity at baseline were associated with a subsequent decrease in agreeableness scores. In multivariable models, these two parameters explained 11% and 8% of variance respectively. The other personality factors were not associated with AD and cerebrovascular imaging markers. **Conclusions**: Personality patterns at baseline may impact the emergence of AD pathology but they may also change rapidly as a function of the presence of early AD-related PET abnormalities.

## 1. Introduction

Personality, long regarded as a relatively stable psychological structure, has recently attracted renewed interest in aging neuroscience due to its sensitivity to biological and cognitive changes. The Five-Factor Model (Big Five), empirically validated across many cultures, describes the personality construct according to five fundamental dimensions: neuroticism, extraversion, openness, agreeableness, and conscientiousness [1]. Recent longitudinal studies have shown that even in the absence of cognitive deficits, levels of extraversion, openness, and conscientiousness tend to decline with age, while neuroticism follows a slightly upward trajectory [2]. These changes are not solely explained by psychosocial adjustments but could also reflect the evolution of brain aging processes. A growing body of evidence has revealed significant associations between certain personality traits and cognitive decline in normal aging. High levels of neuroticism and low levels of conscientiousness are associated with increased cognitive vulnerability, while extraversion and openness may offer some degree of protection [3,4,5,6]. A recent systematic review by Troisi and colleagues [7] also supported the idea that higher levels of conscientiousness and openness may limit the age-related cognitive decrement in healthy controls. Lower agreeableness at baseline was related to increased risk for cognitive decrement but this association was less robust [8].

The relationship between changes in personality assessed with the Five-Factor Model and subsequent development of cognitive impairment in cognitively preserved elderly persons is still disputed. In the Baltimore Longitudinal Study of Aging, no evidence for preclinical changes in personality before the onset of mild cognitive impairment or dementia was identified [9]. In another longitudinal study of a genetically enriched cohort of healthy controls, Caselli and collaborators [10] found that an increase in neuroticism and decrease in openness levels characterize cases that transition to mild cognitive impairment (MCI).

Some neuroimaging studies have examined how personality traits may relate to Alzheimer’s disease (AD) and vascular signature biomarkers, such as loss of brain volumes, cerebrospinal fluid (CSF) Aß and tau levels, positron emission tomography (PET) amyloid deposits and tau pathology, cerebral glucose metabolism, as well as white matter hyperintensities (WMH). In early studies, higher neuroticism was associated with smaller regional volumes and greater decreases in volume with increasing age in individuals without dementia. In contrast, higher conscientiousness was related to larger regional volumes and less decline with advancing age [11]. The positive role of high levels of conscientiousness and openness on AD-signature magnetic resonance imaging (MRI) quantitative markers was also detected in a more recent study by our group [12]. Along the same lines, increasing neuroticism and decreasing extraversion, openness and conscientiousness were associated with lower cerebrospinal fluid (CSF) Aβ1-42 but not with tau and ptau-181 concentrations in mixed cohorts [13]. Several cross-sectional investigations focused on the associations between NEO-PI factors and PET amyloid and tau burden as well as fluorodeoxyglucose (FDG)–PET metabolic patterns prior to cognitive decline. Higher levels of extraversion and conscientiousness were related to lower accumulation of brain beta-amyloid deposition in cognitively normal elderly individuals [14,15]. In the PREVENT-AD cohort including individuals without dementia who have a family burden of AD, lower neuroticism and higher measures of openness and extraversion were related to lower PET amyloid and tau burden [16]. Increased neuroticism was also associated with pathological tau accumulation in regions known to be vulnerable to AD pathophysiology in cognitively normal individuals [17,18]. Consistently, higher neuroticism and lower scores for extraversion and conscientiousness were significantly associated with decreased glucose metabolism in brain regions typically affected by AD neuropathological processes in individuals without dementia [19]. However, negative or ambiguous data were also reported for both amyloid burden and WMH [20,21].

Besides their cross-sectional design, there are two major limitations of these studies. First, personality factors were considered as stable constructs in the course of brain aging. However, several lines of evidence indicate that NEO-PI factor changes occur in the preclinical phases of AD [10,22]. Studying the association between AD imaging markers at baseline and subsequent changes in personality factors may provide arguments related to the hypothesis that personality changes could be the consequence of incipient AD and cerebrovascular pathology. Second, most of the previous studies used quantitative PET markers that are rarely used in busy, routine clinical settings. Our study adopts a 4.5-year longitudinal design focusing on changes in personality traits, assessed by the NEO-PI-R—an established instrument for evaluating the five major personality factors—among older adults without any cognitive impairment at baseline. This assessment is paired with multimodal neuroimaging data, including amyloid PET, FDG-PET, and structural MRI scans. Amyloid positivity and abnormal PET patterns were assessed visually by fully trained specialists blind to cognitive data in order to resemble a real-life situation. The main objective was to explore whether changes in personality traits upon follow-up may be related to the early presence of AD-related abnormal PET and MRI patterns and subtle cognitive changes that may be detected in the course of normal aging. We hypothesize that subsequent changes in personality traits, and in particular neuroticism, openness and agreeableness, are associated with greater amyloid burden, cerebral hypometabolism, increased cerebrovascular burden and medial temporal atrophy (MTA) at baseline.

## 2. Materials and Methods

### 2.1. Participants

The study was approved by the local Ethics Committee, and all participants provided written informed consent prior to inclusion. No changes were made to the study procedures after the Ethics Committee’s approval was obtained. Individuals were recruited from an ongoing cohort study on cognitively intact elders, as described in detail previously [23,24,25,26]. Cases with three neurocognitive assessments at baseline, 18 months and 55 months, brain amyloid and FDG-PET as well as 3T MRI at baseline and APOE status were considered. Exclusion criteria included psychiatric or neurologic disorders, sustained head injury, history of major medical disorders (neoplasm or cardiac illness), alcohol or substance abuse, or regular use of neuroleptic, antidepressant or psychostimulant medication, as well as any contraindications to PET or MRI. Because acute vascular events can confound MRI-based measures, additional exclusion criteria comprised untreated hypertension, subtle cardiovascular symptoms, and a history of stroke or transient ischemic episodes. The final sample comprised 58 cognitively intact controls: 34 women and 24 men, mean age: 72.8 ± 3.6 (mean ± SD), ranging from 69 to 87 years.

### 2.2. Personality Assessment

Personality features and dimensions were evaluated at both baseline and the 55-month follow-up visit using the French adaptation of the NEOPI-R [1], a 240-item self-report questionnaire rated on a five-point Likert scale. The instrument assesses the following five personality factors: neuroticism reflects a propensity toward negative affect, including anxiety, hostility, and anger; extraversion captures a tendency toward positive emotions and feelings such as warmth and enthusiasm; openness is the personal inclination to experience and the appreciation of new situations and thoughts with a curious, imaginative and creative attitude; agreeableness describes trust, cooperation, and altruistic tendencies; and conscientiousness reflects reliability, organization, and adherence to rules and moral standards.

### 2.3. Neuropsychological Evaluation

At baseline, all participants underwent an extensive neuropsychological assessment, including the Mini-Mental State Examination (MMSE) [27], the Hospital Anxiety and Depression Scale (HAD) [28], and the Lawton Instrumental Activities of Daily Living (IADL [29]). Cognitive assessment covered the following domains: (a) attention, assessed with the Digit–Symbol-Coding test [30] and the Trail Making Test A [31]; (b) working memory, assessed with the Digit Span Forward test (verbal) [32] and the Visual Memory Span/Corsi test (visuo-spatial) [33]; (c) episodic memory, assessed with the RI-48 Cued Recall Test (verbal) [34] and the Shapes Test (visual) [35]; (d) executive functions, assessed with the Trail Making Test B [31], the Wisconsin Card Sorting Test, and the Phonemic Verbal Fluency Test [36]; (e) language, assessed with the Boston Naming Test [37]; (f) visual gnosis, assessed with the Ghent Overlapping Figures test; and (g) praxis, including ideomotor [38] and reflexive [39] praxis, and constructional praxis assessed with the Consortium to Establish a Registry for Alzheimer’s Disease (CERAD) figure copy test [40]. All participants were also evaluated with the Clinical Dementia Rating scale (CDR) [41]. In accordance with the criteria of Petersen et al. [42], participants with a CDR score of 0.5 but not meeting criteria for dementia, and with a score more than 1.5 standard deviations below the age-appropriate mean on any cognitive test, were classified as having MCI and were excluded. Participants who met criteria for neither dementia nor MCI at the last follow-up were classified as cognitively healthy controls.

Cognitive changes over time were quantified using a continuous cognitive score derived from the successive assessments, which incorporates both improvements and declines in individual test performance, as previously reported [43]. For each follow-up visit, cognitive trajectories were calculated by counting the number of tests on which a participant’s z-score performance shifted by 0.5 standard deviation or more relative to baseline in either direction. The overall change in cognition from inclusion to the final follow-up (0, 18 and 55 months post-inclusion) was then obtained by summing the continuous cognitive scores calculated at the two follow-up visits, following the same approach described in our earlier work [43].

### 2.4. MRI Data Acquisition

Structural imaging was performed on a 3T whole-body clinical scanner (Tim Trio; Siemens, Erlangen, Germany). A 3D T1-weighted sequence was acquired with the following fundamental parameters: 256 × 256 matrix; 176 sections; 1 × 1 × 1 mm^3^; TE, 2.3 ms; TR, 2300 ms. Additional sequences (T2WI, susceptibility-weighted imaging, diffusion tensor imaging) were used to rule out incidental brain lesions.

### 2.5. Amyloid PET Acquisition

Fifty-four 18F-Florbetapir (Amyvid) and four 18F-Flutemetanol PET (Vizamyl) data were acquired on 2 different scanners (Siemens BiographTM mCT scanner and GE Healthcare Discovery PET/CT 710 scanner) with differing resolution and platform-specific protocols. Image acquisition began 50 to 70 min post-injection for 18F-Florbetapir and 90 to 120 min post-injection for 18F-Flutemetanol. PET images were reconstructed following the parameters recommended by the ADNI protocol, aimed at increasing data uniformity across the multicenter acquisitions. More information on the different imaging protocols can be found on the ADNI website (https://adni.loni.usc.edu/help-faqs/adni-documentation/, accessed on 28 July 2026).

### 2.6. FDG-PET Acquisition

FDG PET/CT imaging was acquired on a Siemens BiographTM mCT or Vision scanner according to the guidelines of the European Association of Nuclear Medicine (EANM) [44]. Following intravenous injection of 200 MBq of 18F-FDG, a 20 min single-bed emission scan was initiated approximately 30 min later, immediately followed by a CT acquisition used for attenuation correction. This ultra-low-dose brain CT was obtained under standard settings (120 kVp, 20 mAs, 128 × 0.6 collimation, a pitch of 1 and 1 s per rotation).

### 2.7. Visual MR Rating

Brain MRI scans were visually rated by an independent, board-certified neuroradiologist (SH), blinded to the neuropsychological data. Medial temporal atrophy was assessed using the standard MTA scale [45] from 0 (absent) to 4 (severe atrophy). White matter hyperintensity load was rated with the Fazekas scale [46] from 0 (no lesions) to 3 (confluent lesions). Cerebral microbleeds were identified on SWI or SWAN sequences, with only probable CMBs retained; corresponding phase images were analyzed in parallel to discriminate probable CMBs versus micro-calcifications [47]. The total number of CMBs was evaluated.

### 2.8. Visual Amyloid PET Rating

The visual analysis of amyloid PET images was conducted by an independent, board-certified specialist in nuclear medicine (VG) with more than 20 years of experience who was blind to the neuropsychological data. Amyloid PET scans were visually interpreted according to each tracer’s approved reading method. For 18F-Florbetapir, a scan was rated positive when two or more cortical areas (each larger than a single gyrus) showed reduced or absent gray–white matter contrast, or when at least one area showed gray matter uptake clearly exceeding that of the adjacent white matter; a negative scan showed preserved gray–white contrast throughout the cortex, with cerebellar uptake not contributing to the classification [48]. For 18F-Flutemetamol, five predefined cortical regions were assessed independently in each hemisphere—the frontal cortex, posterior cingulate/precuneus, lateral temporal cortex, inferolateral parietal cortex, and striatum— and the scan was considered positive if gray–white matter contrast was reduced or lost in at least one of these regions in either hemisphere [49].

### 2.9. Visual FDG PET Rating

FDG PET reading was performed by the same specialist using visual analysis of the output of an automated voxel-wise comparison with a reference database, as recommended in the guidelines [44] and previously described in detail [50]. Images were assessed for the presence, extent, and severity of an Alzheimer’s disease-like pattern of hypometabolism, defined as reduced glucose metabolism involving the posterior cingulate cortex/precuneus and temporoparietal association cortices—the earliest and most typical findings—with relative preservation of the primary sensorimotor cortex, basal ganglia, thalamus, primary visual cortex, and cerebellum (https://pmc.ncbi.nlm.nih.gov/articles/PMC8803744/, accessed on 28 July 2026).

### 2.10. APOE Epsilon 4 Genotyping

APOE epsilon 4 genotyping was performed as previously described [26]. Participants were then dichotomized by APOE epsilon 4 carrier status, distinguishing epsilon 4 carriers (epsilon 4/epsilon 3 genotype) from non-carriers (epsilon 3/epsilon 3 and epsilon 3/epsilon 2 genotypes).

### 2.11. Statistics

Sex differences in demographic, personality and imaging data were assessed with Chi-2, Mann–Whitney u-test and unpaired t-test depending on the variable distribution. A Benjamini–Hochberg correction for multiple comparisons was applied. Predictors of change in personality factor scores (the dependent variable) were identified using simple and multiple linear regression models, including both binary (sex, APOE epsilon 4 status, amyloid PET positivity, visually abnormal FDG-PET), ordinal (education level, baseline MTA, number of CMBs, and Fazekas scores) and continuous (age, baseline MMSE score, continuous cognitive score corresponding to the longitudinal assessment of cognitive functions) data. All candidate variables were entered into LASSO (least absolute shrinkage and selection operator) linear regression models, aimed at identifying the independent variables most strongly associated with change in each of the five NEOPI-R personality factor scores (dependent variables), avoiding for multiple comparison biases. The significance level was set at *p* < 0.05, and all of the analyses were conducted in Stata release 18.5 (College Station, TX, USA).

## 3. Results

The final sample included 58 individuals (34 females, age range: 69–76 years). Demographic, clinical and imaging data are summarized in Table 1 according to the sex distribution. This analysis revealed a series of significant sex-related differences that persisted after correction for multiple comparisons. There was a significant difference in education levels in favor of men in our sample. In contrast, the cognitive decrement upon follow-up was significantly more pronounced in men compared to women. Moreover, the scores for the agreeableness factor both at baseline and follow-up were higher in women compared to men. At first glance, the present series displayed a subtle cognitive decrement over time as illustrated by the small decrease in the continuous cognitive scores, mainly in men. No case satisfied the MCI criteria upon follow-up, remaining within the range of normal aging. However, a closer analysis of AD-signature biomarkers showed that more than 20% of cases had significant amyloid burden on visual inspection or abnormal FDG patterns compatible with the initial phases of the AD process. Along the same lines, mild or moderate mesial temporal lobe atrophy was found in 76% of cases. In respect to vascular burden, moderate-to-severe Fazekas scores were found in 15.5% of cases. Taken together, these descriptive data indicate that a substantial percentage of our cases display initial AD-compatible neurodegeneration and, to a lesser degree, vascular burden at a preclinical phase.

The mean MMSE score was of 28.6 (1.2) with a median value of 28, confirming the cognitive preservation of our cohort. Total agreeableness scores were significantly higher in women both at baseline and follow-up. No significant sex-related differences were found in respect of the other personality factors. Based on LASSO selection of variables, linear regression models were built for each NEO-PI factor score change. In univariate models, the decrease in openness was related to abnormal FDG PET patterns (regression coefficient: −4.89 [−9.18, −0.59], *p* = 0.026). This association persisted in multivariable models. This single variable explained 15% of the variance in the decrease in openness (Table 2). A stronger association between AD-signature markers and personality factors was found for agreeableness in both univariate and multivariable models (Table 3). In univariate models, lower MMSE scores (regression coefficient: 4.35 [1.12, 7.58], *p* = 0.009) and the presence of amyloid positivity (regression coefficient: −10.13 [−19.44, −0.82], *p* = 0.033) at baseline were associated with a decrease in agreeableness scores. In multivariable models, these two parameters persisted as significant predictors of a decrease in agreeableness, explaining 11% and 8% of variance respectively (Table 3). Importantly, no relationship was found between the other personality factors and AD-signature biomarkers. Along the same lines, vascular imaging markers were unrelated to the evolution of personality factors in this cohort.

## 4. Discussion

Our data reveal that amyloid positivity as well as abnormal FDG-PET at baseline are associated with subtle changes in NEO-PI personality factors in normal aging. In this cohort of highly educated elderly persons, the decrease in openness at 4.5-year follow-up was related to abnormal AD-signature FDG patterns at baseline. Initial MMSE scores and PET amyloid positivity may precede the decrease in agreeableness scores. Importantly, these associations were detected using LASSO linear regression models after controlling for demographic factors and other AD- and vascular signature biomarkers.

From a descriptive viewpoint, the sample-related differences in education and cognitive decrement are consistent with our previous publications with the same cohort. In particular, the better cognitive preservation of women despite their lower baseline level of education is consistent with the work of Montandon and collaborators [50], who reported that male gender is an independent predictor of medial temporal lobe volume loss in old age. Closer to the goal of the present report, the gender-related differences in agreeableness scores agree with previous observations in younger cohorts [51,52].

The association between AD patterns of FDG hypometabolism and decreases in openness scores at follow-up suggests that this personality factor is affected early by the progression of the AD process in healthy controls. This observation should be interpreted in conjunction with a series of previous reports on the positive effects of openness in the preservation of hippocampal and inferior parietal lobe volumes [12,53,54,55], as well as white matter tracts connecting posterior and anterior brain regions and the dorsolateral prefrontal cortex [56,57]. Increased functional connectivity within mesocortical networks has also been described in open people [58]. Higher measures of openness were related to lower PET amyloid and tau burden and higher CSF [13,16]. In the same vein, openness has been associated with reversion from mild cognitive impairment (MCI) to normal cognition [59]. Our findings indicate that in the presence of early AD patterns of hypometabolism, the levels of this personality factor decrease rapidly in healthy controls.

One main finding of the present study is the strong association between the decrease in agreeableness and early amyloid positivity in healthy controls. As a psychological construct, agreeableness refers to the tendency to establish interpersonal relationships without aggressively searching for social acceptance. Agreeable persons are more prone to avoid conflicts, adapt themselves to others’ commitments and adopt a majoritarian viewpoint. Traditionally, high agreeableness in the adult lifespan is thought to be a positive trait of personality that promotes subjective well-being [60], better outcomes in mental health treatments [61], less disengagement coping [62] and less sexually aggressive behavior [63]. A previous study showed that higher levels of agreeableness had a modest but significant protective effect on cognition [64]. To date, most of the cross-sectional studies failed to identify a protective role for this personality factor in respect to AD lesions and vascular burden in old age. In contrast, our previous work has shown that high agreeableness and conscientiousness may paradoxically be associated with more pronounced atrophy in the mesial temporal lobe and regions of the mentalizing network (mPFC, amygdala, precuneus), highlighting the heterogeneity of the relationship between this personality factor and brain integrity [43,54]. In conjunction with these observations, the present results show that agreeableness decreases more rapidly in controls with early amyloid positivity and lower MMSE scores at baseline.

From a clinical viewpoint, our findings support the idea that personality patterns may change rapidly as a function of the presence of early AD-related PET abnormalities. The association between lower MMSE scores at baseline and personality changes should be interpreted with caution since its clinical relevance remains limited. First, the MMSE score is a cognitive screener sensitive to several confounders that remains within the narrow window of normal range in our sample with a median value of 28. Second, the continuous cognitive score that explored in detail the cognitive evolution of our controls, taking into account the entire neuropsychological battery, was not associated with personality changes at follow-up. It is thus likely that subtle differences in MMSE score performance within the normal range among elderly controls may be related to a progressive decrease in their agreeableness rather than to subsequent cognitive decline.

Several limitations should be considered when interpreting these data. First, our cases are not representative of the whole spectrum of brain aging. Despite their recruitment in the community, they display no or very mild vascular morbidities, and very low levels of neuroticism compared to age-adjusted normative values. We therefore cannot exclude that the observed associations may no longer be present when cases with mixed demographic profiles or pathologies are considered. Second, the use of visual ratings of AD positivity and FDG-PET patterns instead of standardized continuous metrics (such as centiloids), as in our previous studies, decreases the statistical power but guarantees that our observations may be reproducible in routine clinical settings. In the same line and in order to remain close to “real-life” situations, visual reading was made by a highly experienced specialist in nuclear medicine. We therefore cannot exclude possible errors that could be controlled via additional reading of another specialist blind to the personality data. However, our set of AD biomarkers does not include PET-tau documentation, CSF specimens and, most importantly, plasma AD biomarkers. We therefore cannot comment on their possible association with the evolution of NEO-PI personality factors in the elderly. Lastly, the combination of all significant predictors allows us to explain a modest percentage of personality factor changes. Although substantial given the marked heterogeneity of normal aging and relatively small sample size, this percentage indicates the presence of additional predictors that may impact the association between personality factors and AD-signature and vascular biomarkers. In this respect, the inclusion of mixed cases with significant vascular pathology and higher levels of neuroticism with in vivo assessment of tau pathology, CSF and AD plasma biomarkers in larger elderly cohorts would be a useful complement in the efforts to get better insight into the biological determinants of personality changes in old age.

## 5. Conclusions

The present findings indicate that early changes in AD-PET-related biomarkers may precede the decrease in openness and agreeableness levels in healthy controls long before the emergence of clinically overt symptoms of cognitive decline.

## Figures and Tables

**Table 1:** Demographic and imaging data in the present sample.

Characteristic	Female	Male	Total	*p*
N	34 (58.6%)	24 (41.4%)	58 (100.0%)	
Age	73.1 (4.0)	72.3 (3.0)	72.8 (3.6)	0.460
Education [years]				0.006
<9	7 (20.6%)	0 (0.0%)	7 (12.1%)	
9–12	17 (50.0%)	8 (33.3%)	25 (43.1%)	
>12	10 (29.4%)	16 (66.7%)	26 (44.8%)	
APOE e4 carriers	7 (20.5%)	5 (20.8%)	12 (20.7%)	0.765
MMSE	28.5 (1.3)	28.7 (1.0)	28.6 (1.2)	0.540
Continuous cognitive score	0.2 (4.0)	−2.2 (3.9)	−0.8 (4.1)	0.028
Amyloid PET positivity	8 (23.5%)	5 (20.8%)	13 (22.4%)	0.808
Abnormal FDG PET	9 (26.5%)	5 (20.8%)	14 (24.1%)	0.621
Right MTA	27 (79.4%)	17 (70.8%)	44 (75.9%)	0.452
Left MTA	26 (76.5%)	18 (75.0%)	44 (75.9%)	0.897
Fazekas score				0.569
Absent	13 (38.2%)	12 (50.0%)	25 (43.1%)	
Mild	16 (47.1%)	8 (33.3%)	24 (41.4%)	
Moderate–severe	5 (14.7%)	4 (16.7%)	9 (15.5%)	
Number of CMBs	0.9 (1.5)	0.7 (1.1)	0.8 (1.3)	0.509
Baseline				
Total (N)	77.9 (20.0)	79.2 (21.2)	78.4 (20.3)	0.819
Total (E)	99.1 (16.4)	106.4 (14.2)	102.1 (15.8)	0.083
Total (O)	114.0 (18.4)	114.6 (17.6)	114.2 (17.9)	0.893
Total (A)	136.4 (14.6)	119.7 (22.3)	129.5 (19.8)	0.001
Total (C)	112.5 (20.0)	121.9 (19.7)	116.4 (20.2)	0.081
Follow-up				
Total (N)	78.2 (25.4)	75.5 (23.9)	77.1 (24.6)	0.687
Total (E)	99.8 (16.9)	105.7 (15.5)	102.2 (16.4)	0.185
Total (O)	111.7 (18.1)	115.9 (18.6)	113.4 (18.3)	0.392
Total (A)	136.6 (12.7)	121.2 (19.4)	130.2 (17.4)	<0.001
Total (C)	114.4 (19.2)	123.8 (19.1)	118.3 (19.5)	0.073

N: neuroticism, E: extraversion, O: openness, A: agreeableness, C: conscientiousness; the numbers in parentheses represent percentages or standard deviation values. Note that the only longitudinally collected data were the continuous cognitive score (that corresponds to the evolution of cognition at 18 and 55 months post-inclusion) and personality factor score. Benjamini–Hochberg adjusted *p* value = 0.0045.

**Table 2:** LASSO (least absolute shrinkage and selection operator) linear regression model-selected variables associated with the openness factor score change (R^2^ = 15.0%). Sex and abnormal FDG PET were treated as binary variables, and education as an ordinal variable. The reference category for education was <9 years. Presence of abnormal FDG PET patterns (score of 1 versus 0) was associated with a decrease in openness factor score.

Independent Variable	Univariate				Multiple		
	Crude Coeff.	95% CI	*p* Value	R^2^	Adjusted Coeff.	95% CI	*p* Value
Male sex	3.56	[−0.23, 7.34]	0.065	6.0	2.62	[−1.32, 6.57]	0.188
Education 9–12 y	3.18	[−0.59, 6.95]	0.096	4.9	1.82	[−2.10, 5.74]	0.355
Abnormal FDG PET	−4.89	[−9.18, −0.59]	0.026	8.5	−4.47	[−8.71, −0.23]	0.039

**Table 3:** LASSO (least absolute shrinkage and selection operator) linear regression model-selected variables associated with the agreeableness factor score change (R^2^ = 36.7%). Age and MMSE score at baseline were treated as continuous variables, education years as an ordinal variable (with reference category < 9 years) and APOE 4 positivity as well as abnormal FDG patterns as categorical variables (score of 0 for the absence). Presence of abnormal amyloid PET patterns (score of 1 versus 0) and lower MMSE score at baseline were associated with a decrease in agreeableness factor score.

Independent Variables	Univariate				Multiple		
	Crude Coeff.	95% CI	*p* Value	R^2^	Adjusted Coeff.	95% CI	*p* Value
Age	−0.64	[−1.74, 0.47]	0.255	2.3	−0.76	[−1.76, 0.24]	0.131
Education 9–12 y	5.37	[−2.67, 13.40]	0.186	3.1	6.53	[−0.71, 13.78]	0.076
APOE 4-positive	6.42	[−3.75, 16.59]	0.211	2.8	5.65	[−3.86, 15.17]	0.238
MMSE at baseline	4.35	[1.12, 7.58]	0.009	11.5	3.76	[0.77, 6.75]	0.015
Amyloid PET positivity	−10.13	[−19.44, −0.82]	0.033	7.8	−12.37	[−20.91, −3.82]	0.005
Abnormal FDG PET	7.64	[−1.59, 16.86]	0.103	4.7	8.21	[−0.16, 16.59]	0.054
Right mesial temporal lobe atrophy	6.60	[−2.68, 15.88]	0.160	3.5	6.00	[−2.56, 14.57]	0.165

## Data Availability

The data that support the findings of this study are available from the corresponding author (P.G.) upon reasonable request.

## Abbreviations

The following abbreviations are used in this manuscript:

AD	Alzheimer’s Disease
APOE	Apolipoprotein E
CDR	Clinical Dementia Rating
CERAD	Consortium to Establish a Registry for Alzheimer’s Disease
CMB	Cerebral Microbleeds
CSF	Cerebrospinal Fluid
EANM	European Association of Nuclear Medicine
FDG	Fluorodeoxyglucose
HAD	Hospital Anxiety and Depression
IADL	Instrumental Activities of Daily Living
LASSO	Least Absolute Shrinkage and Selection Operator
MCI	Mild Cognitive Impairment
MMSE	Mini-Mental State Examination
MRI	Magnetic Resonance Imaging
MTA	Mesial Temporal Atrophy
NEO-PI-R	Neuroticism–Extraversion–Openness Personality Inventory–Revised
PET	Positron Emission Tomography
WMH	White Matter Hyperintensities
